# 468. Associations of Socioeconomic Status and Care Access Measures with COVID-19 Testing in Patients with Breast Cancer

**DOI:** 10.1093/ofid/ofad500.538

**Published:** 2023-11-27

**Authors:** Jincong Q Freeman, Xinyi Li, Yong Gun Lee, Ted O Akhiwu

**Affiliations:** The University of Chicago, Chicago, Illinois; Milken Institute School of Public Health, The George Washington University, Washington, District of Columbia; Rutgers, The State University of New Jersey, New Brunswick, New Jersey; MedStar Union Memorial Hospital, Baltimore, Maryland

## Abstract

**Background:**

Breast cancer patients (BCpts) are vulnerable to COVID-19 infection and hospitalization. COVID-19 testing and early detection are critical but can be hindered by socioeconomic status (SES) and care access measures (CAM)-related barriers. Little is known about how SES and CAM affect COVID-19 testing in BCpts at the national level.

**Methods:**

We analyzed data collected from stage I-IV BCpts in the 2020 National Cancer Database, a joint project of the Commission on Cancer of the American College of Surgeons and the American Cancer Society. Multivariable logistic regression was used to examine the associated SES and CAM with COVID-19 testing.

**Results:**

Of 115,948 BCpts (mean age 61.7 years [SD=13.2]), 75.5% were White, 12.3% Black, 3.8% Asian or Pacific Islander (API), 6.6% Hispanic, and 1.8% American Indian or Alaska Native (AIAN). Overall, 79.3% received a COVID-19 test. Compared with White BCpts, API (adjusted odds ratio [AOR]=1.15, 95% CI=1.05-1.26) or Hispanic (AOR=1.24, 95% CI=1.15-1.34) BCpts were more likely, while AIAN BCpts were less likely (AOR=0.89, 95% CI=0.79-0.99), to be tested. No significant differences in testing were observed between Black and White BCpts (AOR=0.98, 95% CI: 0.93-1.04). Uninsured BCpts were less likely than those insured to receive testing (AOR=0.82, 95% CI=0.72-0.93). BCpts residing in Medicaid non-expansion states had lower odds of testing than those in expansion states (AOR=0.89, 95% CI=0.86-0.92). BCpts with a median household income of < $46,227 (AOR=0.79, 95% CI=0.74-0.83), $46,227-57,856 (AOR=0.82, 95% CI=0.78-0.86) or $57,857-74,062 (AOR=0.82, 95% CI=0.79-0.86) were less likely than those with ≥ $74,063 to get tested. BCpts in urban (AOR=0.81, 95% CI=0.77-0.86) or rural (AOR=0.69, 95% CI=0.61-0.78) areas had lower odds of testing than those in metro areas. Compared with BCpts seeking care at academic/research cancer programs, those at community (AOR=0.71, 95% CI=0.67-0.76) or comprehensive community (AOR=0.79, 95% CI=0.76-0.82) programs were less likely to receive a test.

**Conclusion:**

In this cohort of BCpts, one in 5 were not tested for COVID-19, and they were more likely from a lower SES or to have greater CAM-related barriers. Equitable access to COVID-19 testing is needed to address barriers and better reach this population of BCpts.

**Disclosures:**

**All Authors**: No reported disclosures

